# Associations between diabetes self-management and microvascular complications in patients with type 2 diabetes

**DOI:** 10.4178/epih/e2016004

**Published:** 2016-01-25

**Authors:** Fatemeh Mehravar, Mohammad Ali Mansournia, Kourosh Holakouie-Naieni, Ensie Nasli-Esfahani, Nasrin Mansournia, Amir Almasi-Hashiani

**Affiliations:** 1 Clinical Research Development Unit, Imam Hossein Hospital, Shahroud University of Medical Sciences, Shahroud, Iran; 2 Department of Epidemiology and Biostatistics, School of Public Health, Tehran University of Medical Sciences, Tehran, Iran; 3 Diabetes Research Center, Endocrinology and Metabolism Clinical Sciences Institute, Tehran University of Medical Sciences, Tehran, Iran; 4 Imam Reza Hospital, AJA University of Medical Sciences, Tehran, Iran; 5 Department of Epidemiology and Reproductive Health, Reproductive Epidemiology Research Center, Royan Institute for Reproductive Biomedicine, Academic Center for Education, Culture and Research, Tehran, Iran

**Keywords:** Diabetes self-management, Type 2 diabetes, Microvascular complications, Neuropathy, Retinopathy, Nephropathy

## Abstract

**OBJECTIVES:**

Diabetes is a major public health problem that is approaching epidemic proportions globally. Diabetes self-management can reduce complications and mortality in type 2 diabetic patients. The purpose of this study was to examine associations between diabetes self-management and microvascular complications in patients with type 2 diabetes.

**METHODS:**

In this cross-sectional study, 562 Iranian patients older than 30 years of age with type 2 diabetes who received treatment at the Diabetes Research Center of the Endocrinology and Metabolism Research Institute of the Tehran University of Medical Sciences were identified. The participants were enrolled and completed questionnaires between January and April 2014. Patients’ diabetes self-management was assessed as an independent variable by using the Diabetes Self-Management Questionnaire translated into Persian. The outcomes were the microvascular complications of diabetes (retinopathy, nephropathy, and neuropathy), identified from the clinical records of each patient. A multiple logistic regression model was used to estimate odds ratios (ORs) and 95% confidence intervals (CIs) between diabetes self-management and the microvascular complications of type 2 diabetes, adjusting for potential confounders.

**RESULTS:**

After adjusting for potential confounders, a significant association was found between the diabetes self-management sum scale and neuropathy (adjusted OR, 0.64; 95% CI, 0.45 to 0.92, p=0.01). Additionally, weak evidence was found of an association between the sum scale score of diabetes self-management and nephropathy (adjusted OR, 0.71; 95% CI, 0.47 to 1.05, p=0.09).

**CONCLUSIONS:**

Among patients with type 2 diabetes, a lower diabetes self-management score was associated with higher rates of nephropathy and neuropathy.

## INTRODUCTION

Diabetes is a common chronic disease that poses major health problems worldwide and has been called a silent epidemic by the World Health Organization [1]. The prevalence of diabetes throughout the world, including Iran, is increasing [2]. The reported overall prevalence of diabetes in the US was 5.8% in 2012 [3], while the prevalence of diabetes in Iran was 10.9% in 2011 [4].

Patients with diabetes have a high risk of developing long-term microvascular complications that contribute to considerable morbidity and mortality [5]. Approximately 30% to 45% of patients with type 2 diabetes are affected by microvascular diseases [6].The most important risk factors for these types of complications include poor glycemic control, diabetes duration, hypertension, and dyslipidemia [7]. Glycemic control reduces the risk of microvascular complications, which include retinopathy, nephropathy, and neuropathy [8].

A global consensus has emerged that self-management plays an important role in the care of chronic diseases [9]. Diabetes self-management is a process in which the knowledge, skills, and abilities required for a patient to adequately manage his or her condition are facilitated [10]. Diabetes self-management activities include a range of activities, such as ensuring adequate nutrition, regular physical activity, appropriate medication use, feet care, regularly monitoring blood glucose levels, and maintaining a healthy lifestyle [11].

Previous studies have suggested that individuals with diabetes may not follow recommended guidelines for diet and exercise management [12]. Some studies have found that poor diabetes self-management among diabetes patients led to long-term diabetic complications [13,14]. The purpose of the current study was to examine associations between diabetes self-management and microvascular complications in patients with type 2 diabetes.

## MATERIALS AND METHODS

In this cross-sectional study, 562 patients referred to the diabetes clinic of the Diabetes Research Center of the Endocrinology and Metabolism Research Institute of the Tehran University of Medical Sciences were enrolled in the study between January and April 2014.

The inclusion criteria were being more than 30 years of age and having type 2 diabetes (regardless of level on control or the presence of complications) that had been present for more than five years. Patients were excluded from the study if they were pregnant, had severe and enduring mental health problems, were not primarily responsible for their own care, refused to participate in the study, or were participating in another research study.

The outcomes were the microvascular complications of diabetes, including retinopathy, nephropathy, and neuropathy, as identified from clinical patient records, and the exposure was the diabetes self-management score. A dilated retinal exam was performed by an ophthalmologist in our diabetes and metabolic diseases clinic and the results were noted in the patient’s records. Diabetic neuropathy was diagnosed in our clinic via clinical evaluation (examination of the feet and of the patellar and Achilles reflexes), tactile sensitivity (10-g monofilament test), and thermal sensitivity. Patients with persistent microalbuminuria, macroalbuminuria, and elevated serum creatinine levels greater than 1.5 g/dL were considered to have diabetic nephropathy. According to the American Diabetes Association, microalbuminuria is defined as an albumin-to-creatinine ratio between 30 mg/g and 300 mg/g, and macroalbuminuria is defined as an albumin-to-creatinine ratio of 300 mg/g or higher [15].

In order to measure diabetes self-management, we used the Diabetes Self-Management Questionnaire (DSMQ). The DSMQ is a reliable and valid instrument that enables an efficient assessment of self-care behaviors associated with glycemic control [16]. Forward and backward translation techniques for the translation and cultural adaptation of the questionnaire into the Persian language were carried out. The test-retest reliability of the scale was assessed using the intra-class correlation coefficient.

The self-reported questionnaire consists of 16 items divided into four subscales. The first subscale evaluates glucose management, and is scored by items 1, 4, 6, 10, and 12 of the questionnaire. The second subscale addresses dietary control and is scored by items 2, 5, 9, and 13. The third subscale evaluates physical activity and is scored by items 8, 11, and 15 of the questionnaire, while the fourth subscale evaluates healthcare use and is scored by items 3, 7, and 14 of the questionnaire. A sum scale score was derived as a global measure of self-care. The patient’s agreement with each item was scored using a four-point Likert-type scale, ranging from 0 (does not apply to me) to 3 (applies to me very much). Patient records were examined for information regarding demographic information (sex, age, body mass index [BMI], ethnicity, marital status, and education), current diabetes treatment (use of oral hypoglycemic agents and insulin), the duration of diabetes, and the presence of complications of diabetes. The protocol was approved by the Human Subjects Committee of the Tehran University of Medical Sciences. Written consent was obtained from all patients before enrollment.

### Statistical analysis

The quantitative and qualitative data are presented as mean values (standard deviation [SD]) and frequency (percentage), respectively. A multiple logistic regression model was used to estimate the odds ratios (ORs) and 95% confidence intervals (CIs) between diabetes self-management (healthcare, diet, physical activity, blood glucose) and the microvascular complications of type 2 diabetes (neuropathy, nephropathy, and retinopathy), adjusting for potential confounders (age, sex, diabetes duration, hemoglobin A1c [HbA1c] levels, BMI, dyslipidemia, history of coronary heart disease, and hypertension). Given the cross-sectional design of the study, these multiple logistic regression analyses did not compare patients with only one microvascular problem to patients without any microvascular complications. Thus, multiple linear regression analysis was also performed, with the management of diabetes as the outcome variable and diabetic complications as predictor variables. Stata version 12 (STATA Corp., College Station, TX, USA) was used for all statistical analyses.

## RESULTS

Of 600 patients who were initially screened, 562 (93.7%) were enrolled in the study; 38 subjects were excluded because they were pregnant, were not primarily responsible for their own care, or refused to participate in the study. The test-retest reliability was excellent, with intra-class correlation coefficient values of 0.80, 0.72, 0.80, and 0.81 for the glucose management, dietary control, physical activity, and healthcare use subscales of the questionnaire, respectively. The Cronbach’s alpha coefficient value was 0.72, demonstrating adequate internal consistency of the questionnaire.

The mean (SD) age of the participants was 61.62 (10.49) years, with a range of 32 to 89 years. Of the 562 subjects, 232 (41.3%) were male. Of the 562 patients, 264 (47.0%) had no significant complications of diabetes. The prevalence rates of retinopathy, neuropathy, and nephropathy as complications of diabetes were 28.1%, 17.4%, and 14.2%, respectively. The characteristics of the patients, both overall and subclassified according to the presence of microvascular complications of diabetes are presented in Table 1. The mean (SD) of the DSMQ scores is presented in Table 2. However, we did not find any relationship between HbA1c levels and diabetes self-management, as showen in Table 3.

As shown in Table 4, a significant association was found between the sum scale scores of diabetes self-management and neuropathy (unadjusted OR, 0.63 for a one-point increase in the sum scale score of diabetes self-management; 95% CI, 0.44 to 0.91, p=0.01). Scores on the glucose management subscale of the DSMQ were significantly associated with nephropathy (p=0.02) and neuropathy (p=0.03).

After adjusting for potential confounders, significant associations were found between the presence of neuropathy and the diabetes self-management sum scale (p=0.01), the glucose management subscale (p=0.03), and the healthcare use subscale (p=0.02).

The multiple linear regression analysis provided in Table 5 confirmed the results of the multiple logistic regression analysis presented in Table 4.

## DISCUSSION

In this study, we sought to assess associations between diabetes self-management and the microvascular complications of diabetes in patients with type 2 diabetes.

The main results of our study were as follows. First, the mean sum scale of the DSMQ was 4.08 overall (range, 0.83 to 9.17), and the mean sum scale of the DSMQ in patients with retinopathy was better than in patients with nephropathy or neuropathy. Second, lower diabetes self-management scores were associated with microvascular complications in patients with type 2 diabetes. Lower glucose management scores were associated with a higher prevalence of nephropathy and neuropathy. Inadequate healthcare use was associated with a higher prevalence of neuropathy. Diabetes self-management, measured as the sum scale, was inversely associated with the prevalence of neuropathy. The review study carried out by Fritschi & Quinn [17] found many of the chronic complications associated with diabetes to be associated with fatigue. Successful self-management has been inversely associated with reported fatigue levels in chronic diseases, including diabetes and hypertension. Such findings additionally suggest that daily self-management tasks are necessary for maintaining optimal health [18]. Boulton [19] found that lifestyle management and glucose control could diminish the prevalence of idiopathic neuropathy in type 2 diabetes patients. Determining associations between lifestyle factors, including diet and physical activity, and the complications of diabetes was one of the goals of the Japan Diabetes Complications Study [20], and Sone et al. [21] reported that the effect of lifestyle management on improving the glycemic control of patients with established type 2 diabetes mellitus was small but significant three years after initiation of the intervention. Data regarding differences in the occurrence of microvascular or macrovascular complications were not available at that time.

Although some evidence was found of an inverse association between diabetes self-management and nephropathy, we found no associations between diabetes self-management and retinopathy. Our results may be explained by the fact that unlike diabetic neuropathy, diabetic nephropathy and retinopathy are associated with certain unmanipulable factors, such as genetic susceptibility. In our study, as presented in Table 3, we did not identify any relationship between HbA1c levels and diabetes self-management. Heisler et al. [22] demonstrated that HbA1c knowledge was not associated with respondents’ self-efficacy in diabetes care or reported self-management behaviors. Knowing one’s most recent HbA1c level was associated both with accurately assessing one’s level of diabetes control and with reporting a better level of understanding of diabetes care. However, in a 2012 cross-sectional survey, self-management and complications were found to be related to HbA1c levels [23].

Our study found that the management of complications of diabetes was inversely associated with diabetic neuropathy. This finding was consistent with the results of Laxy et al. [24] and Kent et al. [25] who found a negative correlation between scores reflecting the self-management of diabetes and the risk of diabetic neuropathy. Our study showed that blood glucose control, but not other components of self-management, were associated with diabetic nephropathy. Laxy et al. [24] found no association between high scores on an instrument measuring the management of diabetes and microalbuminuria.

In our study, no association was found between the self-management of diabetes and diabetic retinopathy. This finding was inconsistent with the result of the study by Li et al. [26] which showed diabetic retinopathy to be inversely associated with physical activity and dietary control. In sum, our study suggests that among patients with type 2 diabetes, lower diabetes self-management scores are associated with higher rates of nephropathy and neuropathy. The cross-sectional design did not allow us to ascertain whether diabetes self-management affected the microvascular complications of diabetes, making the results prone to reverse causality [27].

The primary source of biases and limitations in cross-sectional studies is the temporal relationship between the exposure and outcome variables [28]. Therefore it is impossible for us to determine if a lower diabetes self-management score was present before the onset of the complications of diabetes or vice versa. However, cross-sectional studies can determine the possible risk factors for the outcome of interest. Cohort or population-based case-control studies can be conducted to ascertain the temporal order of exposure and disease. Another limitation of the present study is that the standard assessment of the DSMQ involves two sets of measurements: one at baseline and a follow-up measurement.

## Figures and Tables

**Table 1.** Patient characteristics, both overall and according to the presence of the microvascular complications of diabetes

Characteristics	Total (n = 562)	Nephropathy (n = 80)	Neuropathy (n = 98)	Retinopathy (n = 159)
Age (yr)	61.62±10.49	64.0±9.43	63.84±9.79	63.93±9.65
Body mass index (kg/m^2^)	27.85±4.38	28.45 ±4.28	27.64±4.26	27.67±4.84
Sex				
Female	330 (58.7)	34 (42.5)	55 (56.1)	88 (55.3)
Male	232 (41.3)	46 (57.5)	43 (43.9)	71 (44.7)
Marital status				
Single	17 (3.0)	3 (3.8)	6 (6.1)	4 (2.5)
Married	451 (80.2)	66 (82.5)	70 (71.4)	135 (84.9)
Widowed	80 (14.2)	7 (8.8)	16 (16.3)	19 (11.9)
Divorced	14 (2.5)	4 (5.0)	6 (6.1)	1 (0.6)
Ethnicity				
Persian	357 (63.5)	48 (60.0)	61 (62.2)	98 (61.6)
Turkish	163 (29.0)	24 (30.0)	27 (27.6)	50 (31.4)
Other	42 (7.5)	8 (10.0)	10 (10.2)	11 (6.9)
Education				
Illiterate	62 (11.0)	11 (13.8)	16 (16.3)	25 (15.7)
High school or less	387 (68.9)	53 (66.2)	65 (66.3)	110 (69.2)
College education	113 (20.1)	16 (20.0)	17 (17.3)	24 (15.1)
Diabetes duration (yr)	12.82 ± 6.61	14.81 ±6.63	14.35±6.62	15.64±6.82
Treatment regimen				
Exclusively oral hypoglycemic agents	292 (52.0)	28 (35.0)	33 (33.7)	57 (35.8)
Exclusively insulin	7(1.2)	1 (1.2)	65 (66.3)	3 (1.9)
Medication and insulin	263 (46.8)	51 (63.8)	0 (0.0)	99 (62.3)
Family history of diabetes				
Yes	333 (59.3)	51 (63.8)	57 (58.2)	96 (60.4)
No	229 (40.7)	29 (36.2)	41 (41.8)	63 (39.6)
Smoking				
Yes	46 (8.2)	12 (15.0)	11 (11.2)	9 (5.7)
No	469 (83.5)	61 (76.2)	79 (80.6)	131 (82.4)
Ex-smoker	47 (8.4)	7 (8.8)	8 (8.2)	19 (11.9)
Hemoglobin A1c values	7.87±1.46	8.28 ± 1.61	8.16± 1.47	8.11 ±1.44

Values are presented as number (%) or mean±standard deviation.

**Table 2.** The Diabetes Self-Management Questionnaire (DSMQ) self-care activities scores, overall and subcategorized according to the presence of the microvascular complications of diabetes

DSMQ	Total (n = 562)	Nephropathy (n = 80)	Neuropathy (n = 98)	Retinopathy (n = 159)
DSMQ sum scale	4.08±0.65	3.95±0.65	3.93±0.52	4.13±0.66
Glucose management subscale	4.62±1.04	4.28±0.94	4.41±1.00	4.56±1.05
Dietary control subscale	4.64±1.32	4.65±1.42	4.45±1.26	4.77±1.40
Physical activity subscale	3.93±1.18	3.87±1.15	3.91±0.97	3.96±1.17
Healthcare use subscale	2.61±1.42	2.52±1.39	2.32±1.17	2.77±1.41

Values are presented as mean±standard deviation.

**Table 3.** DSMQ self-care activity scores depending on HbA1c values

DSMQ	HbA1c (%)			p-value
	≤7.5	7.6-8.9	≥9.0	
DSMQ sum scale	4.05±0.65	4.11±0.65	4.11±0.67	0.55
Glucose management subscale	4.63±1.06	4.56±1.03	4.67±1.02	0.63
Dietary control subscale	4.64±1.35	4.60±1.31	4.69±1.26	0.86
Physical activity subscale	3.90±1.15	4.04±1.25	3.84±1.13	0.31
Healthcare use subscale	2.47±1.27	2.76±1.51	2.71±1.59	0.07

Values are presented as mean±standard deviation. DSMQ, Diabetes Self-Management Questionnaire; HbA1c, hemoglobin A1c.

**Table 4.** Crude and adjusted associations between diabetes self-management and the microvascular complications of diabetes

Variable	Nephropathy		Neuropathy		Retinopathy	
	Crude OR (95% CI)	Adjusted^1^ OR (95% CI)	Crude OR (95% CI)	Adjusted^1^ OR (95% CI)	Crude OR (95% CI)	Adjusted^1^ OR (95% CI)
Glucose management	0.68 (0.53, 0.86)^*^	0.71 (0.55, 0.91)	0.79 (0.63, 0.98)^*^	0.78 (0.63, 0.98)^*^	0.92 (0.77, 1.10)	0.92 (0.77, 1.10)
Dietary control	1.00 (0.84, 1.20)	1.00 (0.83, 1.21)	0.87 (0.73, 1.03)	0.87 (0.73, 1.03)	1.11 (0.96, 1.27)	1.09 (0.95, 1.25)
Physical activity	0.94 (0.77, 1.16)	0.96 (0.77, 1.18)	0.97 (0.81, 1.17)	0.98 (0.81, 1.19)	1.03 (0.88, 1.20)	1.03 (0.88, 1.21)
Healthcare use	0.94 (0.80, 1.12)	0.94 (0.79, 1.12)	0.82 (0.69, 0.97)^*^	0.82 (0.69, 0.97)^*^	1.11 (0.98, 1.25)	1.11 (0.98, 1.27)
Sum scale	0.69 (0.47, 1.02)	0.71 (0.47, 1.05)	0.63 (0.44, 0.91)^*^	0.64 (0.45, 0.92)^*^	1.18 (0.89, 1.56)	1.17 (0.88, 1.56)

OR, odds ratio; CI, confidence interval.

1 Adjusted for age, sex, diabetes duration, hemoglobin A1c, body mass index, dyslipidemia, history of coronary heart disease, and hypertension.

* p<0.05.

**Table 5.** Multiple linear regression for the sum scale score of diabetes self-management^1^

	Coefficient	95% CI	p-value
Nephropathy	-0.13	-0.29, 0.02	0.09
Neuropathy	-0.17	-0.31, -0.02	0.01
Retinopathy	0.07	-0.04, 0.18	0.24

CI, confidence interval.

1 Adjusted for age, sex, diabetes duration, hemoglobin A1c, body mass index, dyslipidemia, history of coronary heart disease, and hypertension.
